# Sudden Unexpected Death and Alcohol Addiction: Case Report on Disulfiram Use

**DOI:** 10.15388/Amed.2025.32.1.5

**Published:** 2025-02-18

**Authors:** Gabija Kondratavičiūtė, Dalius Banionis, Sigitas Laima, Sigitas Chmieliauskas, Diana Vasiljevaitė, Paulius Petreikis, Robertas Badaras

**Affiliations:** 1 Faculty of Medicine, Vilnius University, Vilnius, Lithuania; 2 Department of Pathology, Forensic Medicine, Institute of Biomedical Sciences, Faculty of Medicine, Vilnius University, Vilnius, Lithuania; 3 Department of Anatomy, Histology and Anthropology, Institute of Biomedical Sciences, Faculty of Medicine, Vilnius University, Vilnius, Lithuania; 4 Centre for Toxicology, Clinic of Anaesthesiology, Reanimatology and Critical Care Medicine, Institute of Clinical Medicine, Faculty of Medicine, Vilnius University, Vilnius, Lithuania

**Keywords:** forensic science, disulfiram, autopsy, toxicology, pathology, teismo medicina, disulfiramas, autopsija, toksikologija, patologija

## Abstract

**Background:**

**:** Disulfiram is a type of medication widely prescribed in order to cease alcohol consumption. When used with alcohol, the ‘disulfiram-alcohol reaction’ occurs, causing nausea and vomiting. Disulfiram blocks alcohol dissolving enzymes thus increasing acetaldehyde concentration and inducing the above-mentioned symptoms. These undesirable symptoms are similar to the side effects that occur to oncological patients after the administration of chemotherapy.

**Materials and methods:**

**:** Descriptive research method was used. The literature focusing on alcohol, medications and drug consumption was reviewed.

**Case report:**

**:** The primary abstinence at the beginning of treatment with *Disulfiram* does not guarantee that alcohol usage will not get manifested again at the further stages of treatment. Alcohol addiction forces patients to search for a way to avoid the unwanted reactions. Sometimes, patients tend to be quite ingenious while trying to suppress the arising side effects. In many cases, it involves the usage of additional medication.

**Conclusions:**

**:** When alcohol addiction overpowers, the patient tries to reduce the undesirable symptoms and may even die if alcohol is combined with disulfiram. Death may occur due to the cardio and neurotoxic effects of acetaldehyde.

## Abstract (it)

**Įvadas:**

Disulfiramas – vaistas, plačiai skiriamas priklausomybei nuo alkoholio gydyti. Vartojant šį vaistą kartu su alkoholiu, gali būti disulfiramo ir alkoholio reakcija. Disulfiramas blokuoja alkoholį skaidančius fermentus, dėl to padidėja acetaldehido koncentracija ir sukeliamas pykinimas ir vėmimas. Šie nepageidaujami simptomai panašūs į šalutinį poveikį, kuris būna onkologiniams pacientams po chemoterapijos.

**Medžiaga ir metodai:**

Naudotas aprašomasis metodas. Atlikta literatūros alkoholio, vaistų ir narkotikų vartojimo temomis apžvalga.

**Atvejo pristatymas:**

Pirminis susilaikymas nuo alkoholio gydymo disulfiramu pradžioje negarantuoja, kad alkoholio nebus vartojama tolesniuose gydymo etapuose. Priklausomybė nuo alkoholio verčia pacientus ieškoti būdų išvengti nepageidaujamų reakcijų. Stengdamiesi nuslopinti atsirandančius šalutinius poveikius, jie dažnai ima vartoti papildomų vaistų ir medžiagų.

**Išvados:**

Kai priklausomybė nuo alkoholio pranoksta valią, asmuo bando sumažinti nepageidaujamus simptomus ir gali net mirti, jei derina alkoholį su disulfiramu. Mirtis ištinka dėl toksinio acetaldehido poveikio širdies ir nervų sistemoms.

## Introduction

Alcohol is a widely used psychoactive substance that is legal in many countries and has a long history of consumption and abuse [1]. At first, regular consumption of small amounts of alcohol later may escalate into full-scale alcohol addiction. This happens due to the fact that alcohol consumption provides psychological satisfaction, which later induces larger amounts of alcohol being consumed. Thus, it evolves into psychological and physical addiction [2]. Emerging from cellular and molecular mechanisms, associative memory formation is most likely to act as one of the drivers for persistent drug use via modulatory input from midbrain dopaminergic neurons to multiple circuits in the forebrain [3]. *Disulfiram* (DSF) is used in order to cease alcohol consumption. DSF helps treat alcohol addiction, enabling patients to stop an inadequately high-amount frequent ethanol consumption [1–6]. DSF may be used in two main ways: orally as tablets, or it can be implanted under the skin. Another route of administration, injections, is not widely used for alcohol addiction treatment due to their lack of approval in the European Union (EU) and other national registries, because of a high incidence of severe side effects, and limited clinical support [7]. An implant is strongly suggested, as patients with alcohol addiction tend to simulate the use of medication [8,9]. The implanted DSF provides an even and long-term effect, which is induced depending on the amount of alcohol being consumed [12]. The research regarding implanted DSF showed that, after 6–24 months of treatment, 60–70% of patients undergoing this course of treatment achieved full abstinence. This positive therapeutic effect made it possible for DSF implants to become a highly desirable way of treatment in Europe [8–10].

### DSF’s mechanism of action

DSF blocks the ordinary alcohol metabolism in human organism [1]. The effect of DFS appears immediately when alcohol has been consumed. When ethanol is being consumed, DSF acts through its metabolite, called S-methyl N, N-diethyl-dithiocarbamate-sulphoxide, which blocks ethanol dissolving enzyme aldehyde dehydrogenase (ALDH) [4,5,22]. Due to DSF, alcohol is not fully metabolized, thus resulting in an elevated acetaldehyde concentration. When a patient undergoing DSF treatment consumes a small amount of alcohol, the concentration of acetaldehyde is 5–10 times bigger if compared with the ordinary ethanol metabolism. The accumulation of acetaldehyde causes a complex of specific side-effects called ‘the disulfiram-alcohol reaction’. The onset of these symptoms is proportional to the consumed amount of alcohol and the dose of DSF. DSF with a small amount of ethanol causes headaches, vertigo, skin flush, perspiration, eyesight disorders, hypertension, tachycardia, nausea, vomiting, and dyspnea. A larger amount of alcohol causes cardiotoxic and neurotoxic effects due to an elevated acetaldehyde concentration, and it may result in respiratory distress, myocardial infarction, unconsciousness and seizures. Large amounts of ethanol may lead to death [1,2,5].

DSF is removed from the organism by reducing it into a metabolite called diethyldithiocarbamate (DDC). DDC is formed after DSF reacts with glutathione reductase protein [13]. DDC is further eliminated by reducing it into dimethylamine and carbon disulfide, with the latter substance being exhaled with CO_2_. An alternative elimination path includes DDC methylation forming S-Methyl N,N-diethyldithiocarbamate (MeDDC). MeDDC is further oxidized into sulphide and sulfoxide which are excreted with urine. The blood concentration of DDC and MeDDC varies from 0.48 µmol during the first day, and down to 0.11 µmol on the third day [10,12].

The symptoms of the disulfiram-alcohol reaction vary from patient to patient. A thorough instruction of the patient is necessary, as the varying side effects are hard to forecast. The manifestation of the DSF-alcohol reaction stops when the administration of DFS is ceased. However, the recovery of ALDH activity is usually completed after a week. Thus, patients should not consume any amount of alcohol for the 2 weeks after the end of treatment by DSF.

Substances that may cause disulfiram-like reactions (DLR) when combined with ethanol include certain mushrooms, such as *Coprinus atramentarius* (inky cap), which was once considered a potential treatment for alcoholism [15]. This mushroom contains the protoxin coprine (N5-1-hydroxycyclopropyl-L-glutamine), which, after ingestion, is converted to 1-aminocyclopropanol. This metabolite inhibits the enzyme ALDH, blocking alcohol metabolism similarly to disulfiram. However, coprine was ultimately deemed unsuitable for therapeutic use due to its mutagenic, gonadotoxic, and DLR effects [16].

Additional agents known to provoke DLR include industrial solvents (referred to as “degreasers’ flush”), antifungals like griseofulvin, pesticides such as carbamates and monosulfiram, and chloral hydrate [17]. Another well-known reaction occurs between metronidazole and alcohol, which was previously thought to produce a disulfiram-like reaction. However, research questions the strength of evidence for this interaction, suggesting that it may be overstated, and it likely results from factors other than a true disulfiram-like biochemical mechanism [18].

## Materials and methods

The literature focusing on alcohol, medications and drug consumption was reviewed. A circumstantial search was conducted across the *PubMed* database and the online *Google Scholar* search engine by using keywords relevant to the subject, including ‘disulfiram’, ‘alcohol’, ‘treatment’, ‘alcoholism’, and ‘drug therapy’. The search involved articles published in the English language, and a total of 30 pertinent articles have been selected for inclusion in the literature review. Additionally, associated information to the analysis of clinical case, sourced from the *Lithuanian State Forensic Medicine Service*, has been involved in the examination. During the autopsy, blood and urine samples were systematically collected for alcohol, medications, and drug testing.

## Case report

The body of a 52-year-old man was examined. The corpse was found in the kitchen of the deceased person’s apartment, sitting in the corner, leaning against the wall. A half-empty bottle of vodka and another empty one were found on the table. Next to the bottles, a joint of green, organic substance was placed. No additional information about the health issues of the deceased individual, or the treatments he might have undergone was available. During the external examination, a couple of bruises and a small wound of the occiput were noted. These identified injuries did not have any effect on the cause of death, as they were only superficial. Two 1.5x0.1 cm sized, white, stiff scars were found at the both sides of the lower region of the abdomen. The scars were localized a couple of centimetres above the inguinal wrinkles. After palpation, the scars appeared to be non-migratory, and, underneath them, 3 cm-sized (in diameter) formations were discovered. After separating the skin at the scar projection in the fatty tissue, four pills of an unidentified substance were found at each side. The pills were white, encapsulated in connective tissues, 0.5 cm in diameter, with their edges being uneven. No reaction of the surrounding tissue was evident. During the internal examination, further findings were marked: the heart weighted 455 g, the coronary arteries were damaged by atherosclerotic plaques narrowing the lumen up to 75%, there were stones in the gallbladder, and signs of sudden death were evident. The toxicology tests revealed 1.95 ‰ (195 mg / 100 ml) of blood ethyl alcohol and 3.59 ‰ (359 mg / 100 ml) of ethyl alcohol in urine. Furthermore, 2000 ng/ml of citalopram, and 240 ng/ml of tetrahydrocannabinol acid were found in the urine. 0.06 mg/L of disulfiram and traces (81 ng) of citalopram were found in blood. The removed pills were identified as disulfiram. The cause of death was an acute, toxic effect of the interaction of ethanol, medications, and drugs.

## Discussion

Implantation of disulfiram under the skin protects the patient undergoing the treatment from simulation. It also provides an even distribution of the medication. The initial ‘disulfiram–ethanol reaction’ side-effects may manifest when the blood alcohol concentration is 5–10 mg / 100 ml. When the alcohol concentration reaches 125–150 mg / 100 ml, the patients are usually unconscious. In case of an acute toxic alcohol effect, when the blood ethanol concentration reaches 200 mg / 100 ml, motor dysfunctions are common. Furthermore, muscle response to stimulation may become absent, muscle synergism gets disrupted, and cardiogenic death takes place [12–13].

Based on the producer’s recommendations, when a DSF implant is used alone, it is conditionally perceived as a non-toxic substance. The recommended, safe and effective dose of implanted DSF is 400–1200 mg. An indicated average duration of treatment is 3–6 months. DSF absorption from an implant is constant and even. Patients should receive a 5.6 mg daily dose of DSF during treatment. Disulfiram is distributed in 125–500 mg pills [10]. Disulfiram implantation is not registered within the European Union; however, surgical implantation of disulfiram into the muscle or subcutaneous tissue may be administered upon the patient’s written request [14]. However, in this particular case, the deceased individual’s medical records did not include any information about the location/institution where and when this medication had been administered.

Long-term consumption of ethyl alcohol causes an unhealthy psychological addition and a need to get intoxicated. When DSF is combined with alcohol, the ‘disulfiram–ethanol reaction’ occurs, which is similar to the side-effects manifesting in oncological patients after chemotherapy, specifically, nausea, vomiting, and vertigo. When treating oncological diseases, cannabis is legalized in medical practice, as it effectively suppresses the presently mentioned symptoms [10]. The therapeutic effect of cannabinoids begins with delta-9-tetrahydrocannabinol (9-THC) being absorbed in the blood. It combines with CB1 – endogenic cannabinoid receptors of the brain and the digestive system, pushing out anandamide and re-stimulating these systems. Anandamide is a natural cannabinoid, found in the human organism, which is believed to exert a significant effect on a person’s eating habits, motivation, sleep, and suppression of pain [27]. Furthermore, it also suppresses motility and has a psychotropic effect [19–22]. THC works as an antagonist to serotoninergic 5-hydroxitriptamin receptors, thus reducing nausea and vomiting [20,21,23]. An intense modulation of CB1 receptors may cause a certain cardiogenic effect, such as a low arterial blood pressure and suppressed contraction of the myocardium [15,19,22,24].

In this case report, the determined blood ethanol concentration was 195 mg / 100 ml. Cannabinoids provided the undesirable reaction suppressing effect, allowing the patient to reach a deadly concentration of ethyl alcohol. The individual’s death occurred during the alcohol elimination phase, as indicated by the higher alcohol concentration in the urine than in the blood, suggesting that the fatal intoxication occurred when the acetaldehyde concentration was at its peak. Cardiogenic death was caused by ethyl alcohol interaction with DSF, during which, an even larger concentration of acetaldehyde was produced. Acetaldehyde is characterized as having a direct cardiotoxic effect on the heart’s conductive system, thus disrupting the heart’s activity [11,25,26].

A literature search on sudden deaths following disulfiram treatment revealed that early high-dose regimens (1000–3000 mg/day) were linked to severe, sometimes fatal reactions when patients consumed alcohol, with cases from the 1950s reporting sudden respiratory or circulatory collapse [28]. Initially, disulfiram treatment involved aversion conditioning, where patients were given a test dose of alcohol after disulfiram to experience its unpleasant effects and discourage drinking. However, after a 1952 U.S. case in which a patient died following alcohol ingestion on high-dose disulfiram, the approach shifted towards patients being verbally informed about the potential reactions rather than getting directly exposed [27,30]. Such fatalities are not documented in the latest literature available to the authors of the present report.

## Conclusions

Because of alcohol addiction, implanting disulfiram under the skin ensures a consistent medication release and helps prevent patient non-compliance, which is a common issue when individuals, often pressured by others, attempt to seek treatment. An attempt to bypass disulfiram-alcohol reactions with cannabinoids or any other substances can be fatal, as they may allow patients to consume high levels of alcohol undeterred by the drug’s effects, leading to toxic concentrations of acetaldehyde. ‘Disulfiram – alcohol reactions’ trigger nausea, vomiting, and tachycardia, and these effects may be deadly to the patient. When the alcohol concentration reaches 125–150 mg / 100 ml, individuals lose consciousness. When the blood alcohol concentration reaches 200 mg / 100 ml, death may occur. Meanwhile, such alcohol concentrations are usually not dangerous from a clinical toxicological point of view, when consumed without DSF. When applying implanted disulfiram treatment, the dangers and results of disulfiram’s reaction with alcohol must be explained to the patient, in case the patient makes an attempt to ignore the doctor’s recommendations. Before starting treatment with implanted DSF, the doctor must evaluate the patient’s cardiological condition because of the possible, direct cardiotoxic effect of acetaldehyde. The patient must receive a series of consultations including a visit to a toxicologist and a psychiatrist. This is necessary for the patient to fully understand the possible negative effects of the DSF treatment regime violations.
